# Genetic profile of domestic cat (Felis catus L.) population of Aoshima Island (Japan)

**DOI:** 10.18699/VJGB-23-23

**Published:** 2023-04

**Authors:** S.K. Kholin

**Affiliations:** Federal Scientific Center of the East Asia Terrestrial Biodiversity, Far Eastern Branch of the Russian Academy of Sciences, Vladivostok, Russia

**Keywords:** Felis catus, genetic profile, islands, founder effect, Japan, генетический профиль, острова, эффект основателя, Япония

## Abstract

The paper analyzes the genetic profile of the domestic cat population of the Aoshima Island. The population has been established in the middle of the last century, after a small group of animals was imported for rodent control. Based on three photographs, the genotypes of the cats in three overlapping groups (75, 56, and 70 individuals) were determined. The mutant allele frequencies of the sex-linked O (Orange) locus and the three autosomal loci a, W, and l (Agouti, White, and Long hair) responsible for coat color and length were estimated. The population lacks the mutant alleles d (Dilution locus), W and wg (White), tab (Tabby), Ti A (Ticked) present in other populations of Japan. This is an almost monomorphic population with prevailing red and tortoiseshell individuals. Most cats have interrupted stripes (genotype Ti+Ti+TaM-). The island’s population differs from the other populations of the Japanese islands in the frequencies of two mutant alleles, O and a. The frequency of the O allele (q(O) = 0.580) is one of the highest in the region, while the frequency of the a allele (q(a) = 0.276) is two times lower than in the other populations. In both cases, the differences in frequencies between the neighbouring populations are significant (p < 0.0001). An independent study of the same population revealed a similar genetic structure. However, it detected the presence of the d allele, the frequency of the a allele was higher (0.534 versus 0.276, p < 0.020). The genetic profile, frequencies of mutant alleles in the population, and history of its origin indicate a significant influence of the founder effect on the genetic structure of the island’s domestic cat population.

## Abstract (ru)

Проанализирован генетический профиль популяции домашней кошки острова Аошима (Япония). Популяция возникла в середине прошлого века в результате завоза небольшой группы животных для борьбы с грызунами. По трем фотографиям определен фенотипический состав групп животных (75, 56 и 70 особей). Рассчитаны частоты мутантных аллелей сцепленного с полом локуса Orange и трех аутосомных локусов (Auguti, White и Long hair), отвечающих за окраску и длину шерсти. В популяции отсутствуют мутантные аллели d (локус Dilution), W и wg (White), tab (Tabby), TiA (Ticked), отмеченные в других популяциях Японии. Фенотипически это практически мономорфная популяция с преобладающим большинством рыжих и черепаховых особей. Большинство кошек имеют прерванные полосы (генотип Ti+Ti+TaM-). Популяция острова резко отличается от остальных популяций островов Японии по частотам двух мутантных аллелей, O и a. Частота аллеля O (q(O) = 0.580) – одна из самых высоких частот в регионе, а аллеля a (q(a) = 0.276) – меньше в два раза, чем в других популяциях. В обоих случаях отличие от частот в окружающих портовых популяциях статистически значимо (p < 0.0001). Генетический состав, частоты мутантных аллелей в популяции и характер ее происхождения указывают на значительное влияние эффекта основателя на генетическую структуру популяции домашней кошки о-ва Аошима. Независимое исследование популяции кошек о-ва Аошима обнаружило сходную генетическую структуру. Однако в популяции найдены особи, носители аллеля d. Возможно, это может быть вызвано различиями в методике определения фенотипа таких кошек. Частота аллеля a статистически значимо выше (0.534 против 0.276, p < 0.020). Все это, однако, не влияет на общий вывод о действии эффекта основателя при возникновении популяции кошек этого острова

## Introduction

The domestic cat is a unique object of genetic research due to the high polymorphism of its populations for a number of traits (color, texture and length of coat, and some other features of external morphology). Freely living in human settlements, cats do not depend on humans for their reproduction. In this sense, they are similar to true natural populations. Cats’ phenotypes can be easily identified at a distance. This makes it possible to collect sufficient data on allele frequencies without much effort. Such data can be used in various kinds of population genetic analysis, e. g., it has been shown that every cat population has its own genetic profile determined by its origin, location and population history (Lloyd, 1987).

From the point of view of population genetics, the history of studying the domestic cat (Felis catus L., 1758) dates back more than 65 years when the first investigation on the frequencies of the mutant alleles responsible for the color and length of coat in London’s cat population was published (Searle, 1949). A surge of interest in the subject occurred in the late 1960s – early 1980s, when the research covered most of Europe and North America, Southeast Asia, and Australia (Kholin, 2018).

There are various methods for obtaining data on mutant allele frequencies in domestic cat populations, each having certain advantages and drawbacks (Twedt, 1983; Schüler, Borodin, 1992). Before the advent of digital cameras, the main method was direct observation of animals on the streets, in the yards or door-to-door survey. Н. Todd and Р. Jeanne (1972) were the first to use the photographic method. The use a single photo of a group of about 100 cats in Sao Paulo (Brazil) to carry out a detailed analysis of the cats’ phenotypes and calculate the frequency of mutant alleles with varying degrees of accuracy. Digital photography has greatly facilitated the collection of the data and made it possible to obtain samples of sufficient size (several images of one individual), to accurately describe a cat’s phenotype in the lab.

In Asia, it is the Japanese domestic cat that has been most intensively studied. Data on 105 (Nozawa et al., 1990), 174 (Nozawa et al., 2000) and 141 (Nozawa, Kawamoto, 2013) cat populations from small villages to megapolises in all prefectures of the four largest and 35 small islands have been obtained (Nozawa, 2019).

One of these islands, Aoshima, is about 400 hectares in size and located off the northern coast of Kyushu Island in the Inland Sea of Japan. The island is currently populated by no more than 10 people and 200 cats https://www.nippon.com/ru/behind/fnn20181019001/?pnum=2. Last accessed March 12, 2023.

Last accessed March 12, 2023.. The cats were brought there in the middle of the last century to reduce the number of rats damaging fishing nets. Eventually, the fishing industry fell into decay, but the cats remained, now being fed by the locals and the tourists arriving on the island.

The aim of the present study was to describe the genetic profile of the island’s domestic cat population by analyzing the photographs of a group of cats and to compare the findings with the previous study of the Aoshima cats (Nozawa, 2019) Unfortunately, the paper does not specify how the data were obtained. It is only known that the observations were carried out before 2015.and other population studies.

## Materials and methods

In March 2015, the photos of the island’s cats were published in the media and on the Internet (Photos by Thomas Peter, Reuters, February 25, 2015).http://www.theatlantic.com/photo/2015/03/a-visit-to-aoshima-a-cat-islandin- japan/386647/. Last accessed March 12, 2023 The quality of the published images was high enough to accurately describe the cat phenotypes. In this study, they were used as samples to assess the genetic profile of the cat population.

The photographs show individual cats and groups on the pier waiting for a boat to arrive. Three photos were selected to contain the largest numbers of animals (see the Figure and Supplementary Material)4. Each photograph was considered a separate sample from the same set of cats to estimate the stability of the assessment of the phenetic (genetic) composition of the population. The sample size for each locus depended on the visibility of each animal in the photograph. In total, the data of 75, 56 and 70 individuals were recorded (sample A, B, and C, respectively). The photographs were used to determine the number of individuals carrying a particular phenotype.

Supplementary Materials are available in the online version of the paper: http://vavilov.elpub.ru/jour/manager/files/Suppl_Kholin_Engl_27_2.pdf.


**Sample ab.**
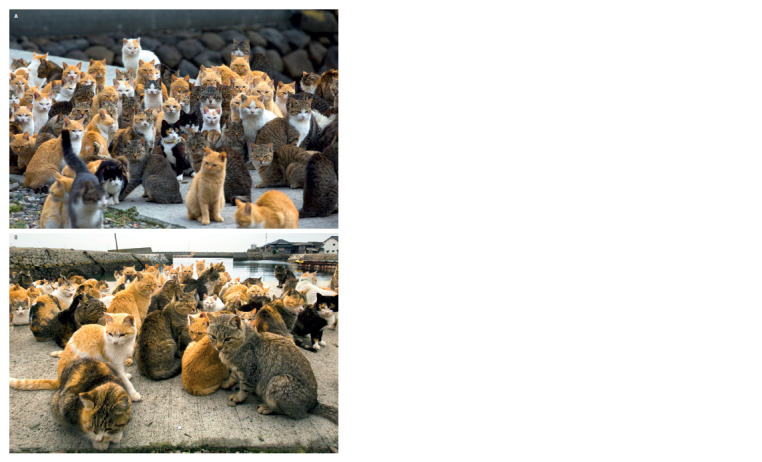
Aoshima cats selected as samples A and B (source: Reuters/Pixstream).

**Sample c.**
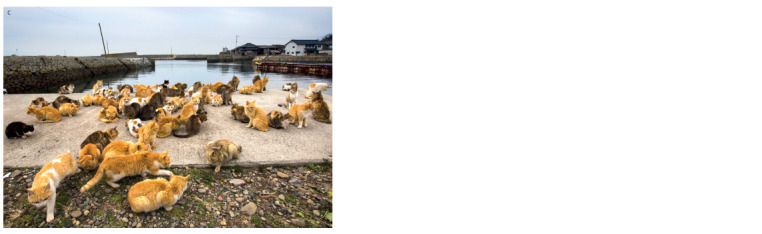
Aoshima cats selected as sample C (source: Reuters/Pixstream)

The data were used to calculate the frequencies of the O allele of the sex-linked Orange locus, as well as the frequencies of two alleles of autosomal loci: Agouti (a) and Long hair (l ) (Table 1). The first two loci control the color of the coat, and the last, its length. The pattern of inheritance of these traits was described by R. Robinson (1993a, b). No individuals carrying the d allele of the Dilution locus were detected in any of the photographs.

**Table 1.**
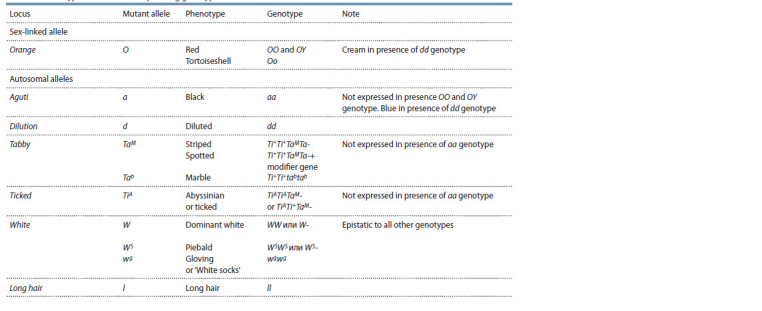
Phenotypes and their corresponding genotypes in the domestic cat

The stripe pattern determined by autosomal loci Tabby (Ta) and Ticked (Ti ) was also analyzed. The inheritance of this trait was described by E. Eizirik et al. (2010). The analysis demonstrated that among the cats there were no individuals homozygous for the tab allele producing marble coloration (Blotched tabby). Most of the cats had interrupted stripes (genotype Ti+Ti+TaM-). The cats carrying the dominant Ti A allele are characterized by a complete absence of stripes on the body and the presence of stripes only on the head, paws and tail. Two individuals (see the Figure 1, B; Supplementary Material), (Nos. 29 and 42) in the foreground have a similar phenotype. However, the Ti A allele was excluded from the analysis due to the difficulty of its unambiguous identification in the available photographs.

Previously, the Piebald white spotting (S) and Dominant white (W ) were considered mutations at different loci. However, modern data indicate that they are semi-dominant (W S ) and dominant (W ) mutations at the same W (KIT ) locus (David et al., 2014). The former is responsible for the piebald coloration, the carriers of the latter are completely white. There is a third, previously unknown, recessive mutation w g (Gloving) producing ‘white socks’ in homozygous cats. None of the examined photos show the cats carrying any of the two last alleles (w g or W ).

The frequencies of recessive alleles (q) were calculated as the square root of the frequencies of the corresponding phenotypes, and of dominant ( p) – as p = 1 – q. The standard errors (SE) were calculated as 1 – q2 4n and p(2 – p) 4n , respectively (Robinson, Manchenko, 1981; Goncharenko et al., 1985). Since the sex of the animals was not determined, the O allele frequencies of the sex-linked Orange locus were estimated using the maximum likelihood method assuming an equal sex ratio (Adalsteinsson, Blumenberg, 1984). In the first approximation, the formula q = 2a + b 2n , was applied where a and b are the numbers of red (genotype O/–) and tortoiseshell (O/+) cats, and n is the sample size (n = a + b + c, where c is the number of nonorange (+/–) individuals) (Robinson, 1972). To get a more accurate estimate an iterative algorithm qi+1 = qi + dL dqi Var(qi) was used, where dL dq = a 1 + q + a + b q – c 2 – q – b + c 1 – q , 1 Var(q) = 0.5N q 1 + q + 3 – q q + 1 – q 2 – q + 2 + q 1 – q . Its SE was calculated as √ Var(q).

To estimate the random mating (panmixia), the expected numerical ratio of genotypes a, b and c was estimated using the formulas: 0.5qn(1 + q), qn(1 – q) и 0.5n(2 – q) (1 – q), respectively.

Testing of the statistical hypotheses was carried out using the χ2- and G-tests, the last having a distribution similar to that of χ2 but being more convenient for analyzing contingency tables. Pairwise comparison of samples for individual loci was carried out using the χ2-test and the arcsine-transformation of the allele frequencies (Zhivotovsky, 1991).

To assess the genetic differentiation of Fst and Gst (Kuznetsov, 2020), a computational add-on for Excel GenAlEx 6.503 (Peakall, Smouse, 2012) was applied.

## Results and discussion

Table 2 shows the results of testing for panmixia at the Orange locus. In all cases we observed a good correspondence between the observed and expected frequencies of the genotypes (p > 0.20). The test for heterogeneity in the ratio of the genotype frequencies indicated the absence of significant differences between the samples for this characteristic (G = 1.232, df = 4, p > 0.85). The O allele frequency in the samples under consideration ranged 0.570–0.589 (mean, 0.580 ± 0.052) and was homogeneous (χ2 = 0.049, df = 2, p > 0.95).

**Table 2.**
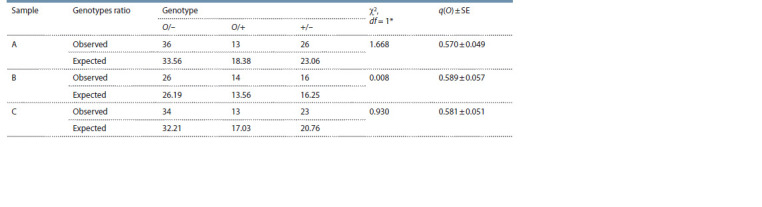
Observed and expected genotype ratios of the Orange locus; the results of the χ2-test for panmixia; and the estimate of O allele frequency (q(O)) in the samples from the Aoshima Island Notе. “–” means the state of the second allele is unknown; * means p > 0.20 in all cases.

Table 3 shows the estimates of the frequencies of the other alleles calculated under the assumption of panmixia. Testing for heterogeneity in the frequencies of mutant phenotypes did not reveal significant differences between the samples ( p > 0.15 in all cases).

**Table 3.**
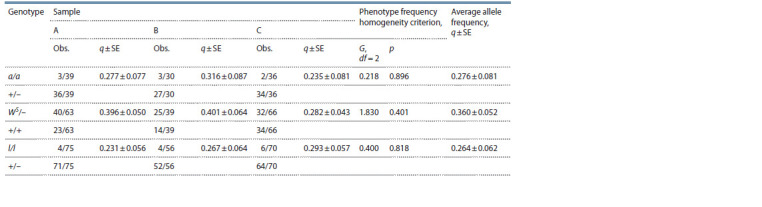
Observed phenotype ratio (Obs.) and mutant alleles frequency estimates (q) in the island’s samples

A comparison with data on the frequencies of mutant alleles in the main islands of Japan showed that the allele Ofrequency (0.580) in Aoshima was two or more times higher than in neighboring populations (q(O) = 0.232 (0.154– 0.412)), and the frequencies throughout Japan (q(O) = 0.220 (0.095–0.490)) (Nozawa, Kawamoto, 2013). In samples B and C, the differences were significant ( p <0.0001). In the case of allele a, the situation was reversed. Its frequency (0.276) was two or more times lower than in the neighboring populations (q(a) = 0.691 (0.614–0.783)) and throughout Japan (q(a) = 0.697 (0.463–0.839)), ( p < 0.0001). Allele l frequency (0.264) fit into the range of variability in the surrounding populations (q(l ) = 0.214 (0.117–0.307)) and all Japanese islands (q(l ) = 0.181 (0–0.412)), ( p> 0.15).

In their studies published earlier (Nozawa et al., 1990, 2000; Nozawa, Kawamoto, 2013) pursued outdated ideas about the genetics of piebald coloration in cats: the piebald cats were considered as carriers of the dominant S allele of a Piebald white spotting locus. However, since the island’s cat population has no w g allele, for ease its comparison against the surrounding populations, in our study a proportion of piebald cats was used. In the considered samples they comprised 63.5 ± 6.1 %, 64.1 ± 7.7 % and 48.5 ± 6.2 %, respectively (mean 58.7 ± 6.6 %). According to the test results, the samples were homogeneous in terms of the frequency of this trait (see Table 3, p = 0.401). The proportion of piebald cats on the island was not much higher than that in the surrounding populations (q = 0.456 (0.346–0.523)) and fit within the variability range for all the Japanese islands (q = 0.532 (0.188–0.815), p > 0.25).

Examining other photos of the island’s cats showed the presence of the so-called bobtail (short-tailed) cats. Unfortunately, the available photos did not allow to estimate their proportion in the population, while in Japan their ratio varied from 0 to 79.6 % (mean 28.8 %) (Nozawa, Kawamoto, 2013).

There are not so many publications (about 40) devoted to the population genetics of island domestic cat populations (Kholin, 2018). In most cases, the mutant allele frequencies of the populations correspond to those in the populations they originated from (Lloyd, 1987). This situation is commonly observed on the islands with stable large settlements of people such as the Azores whose cat population came from Portugal (Todd, Lloyd, 1984). However, there are cases when groups of cats introduced accidentally or deliberately to small islands become feral. In such populations, the original genetic profile of the founding group has been preserved as a result of the founder effect (Dreux, 1974; van Aarde, Robinson, 1980; Jones, Horton, 1984).

This is the case of the Aoshima Island where the cats, once brought to the island, lived their lives protecting fishing nets from rats and nothing has changed for them in this respect since the fishermen left the island. This is why this population has contrasting genetic differences when compared to the nearby populations it may have descended from. This is evidenced by the high genetic differentiation (Table 4) at two loci between the island population and that of their possible ancestors, populating the nearest port of Matsuyama City. This differentiation also indicates the founding group had a homogeneous phenotypic composition. With a greater probability, these were red cats, since among sailors and fishermen there is a belief that red cats bring good luck.

**Table 4.**
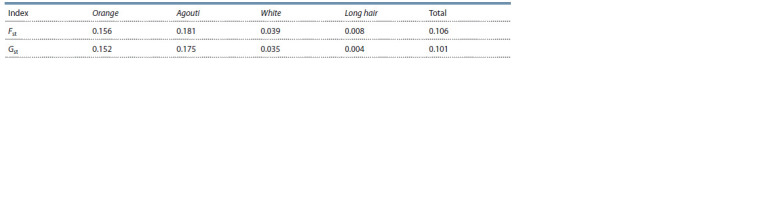
Indices of genetic differentiation between the insular and “mainland” populations

One of the indirect confirmations of the absence of significant migration to the island after the cat population was established is the following fact. In the populations of the main islands of Japan, relatively low frequencies of the d and tab alleles are observed due to the country’s long-term historical isolation. In the postwar years, a steady increase in the proportion of cats carrying these alleles was noted for they became popular with the people of Japan (Nozawa, Kawamoto, 2013). However, in the Aoshima population there are still no cats with the “marble” phenotype homozygous for the tab allele.

The presented data and the results of another study (Nozawa, 2019) indicate the resemblance of the frequency estimates obtained by different observers since in both cases comparable sample sizes have been obtained (56–75 and 72 individuals, respectively). Table 5 shows the estimates of the mutant allele frequencies and the results of their statistical comparison, which indicate no statistically significant differences, except for the a allele, the frequency of which is significantly higher in the (Nozawa, 2019) sample. Another difference is the presence of cats of diluted color phenotype the in the Nozawa (2019) sample and their absence in our samples. This may be due to differences in the method for determining the phenotype of such cats. Thus, the founder effect has played a main role in the formation of the genetic composition of the island cat population

**Table 5.**
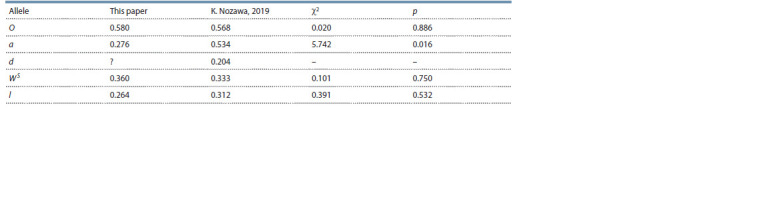
Mutant allele frequencies estimations of Aoshima’s domestic cat population and their statistical comparison

What is interesting is the population’s future since most of the cats were neutered in 2018 https://www.nippon.com/ru/behind/fnn20181019001/?pnum=2. Last accessed March 12, 2023.. But how this will affect the genetic structure of this population and its condition as a whole would only be shown by future research.

## Conclusion

The genetic profile of the domestic cat populating the Aoshima island differs sharply from that of the populations of the port cities surrounding the island, and the Japanese population as a whole. The island’s cats lack the alleles common to other populations, and have one of the highest frequencies of the O allele (q(O) = 0.580) observed in Japan. Phenotypically, this is an almost monomorphic population mainly composed of red and tortoiseshell individuals, which is probably due to the single introduction by fishermen to the island of a small group of cats with a high frequency of O allele carriers. Thus, the founder effect had a large influence on the formation of the genetic composition of the island’s cat population.

## Conflict of interest

The authors declare no conflict of interest.
